# Can quantitative peritumoral CT radiomics features predict the prognosis of patients with non-small cell lung cancer? A systematic review

**DOI:** 10.1007/s00330-022-09174-8

**Published:** 2022-10-29

**Authors:** Linyu Wu, Xinjing Lou, Ning Kong, Maosheng Xu, Chen Gao

**Affiliations:** 1 Department of Radiology, The First Affiliated Hospital of Zhejiang Chinese Medical University (Zhejiang Provincial Hospital of Chinese Medicine), 54 Youdian Road, Hangzhou, China; 2 The First School of Clinical Medicine of Zhejiang Chinese Medical University, Hangzhou, China

**Keywords:** Carcinoma, non-small-cell lung, Solitary pulmonary nodule, Prognosis, Tomography, X-ray computed, Machine learning

## Abstract

**Objectives:**

To provide an overarching evaluation of the value of peritumoral CT radiomics features for predicting the prognosis of non-small cell lung cancer and to assess the quality of the available studies.

**Methods:**

The PubMed, Embase, Web of Science, and Cochrane Library databases were searched for studies predicting the prognosis in patients with non-small cell lung cancer (NSCLC) using CT-based peritumoral radiomics features. Information about the patient, CT-scanner, and radiomics analyses were all extracted for the included studies. Study quality was assessed using the Radiomics Quality Score (RQS) and the Prediction Model Risk of Bias Assessment Tool (PROBAST).

**Results:**

Thirteen studies were included with 2942 patients from 2017 to 2022. Only one study was prospective, and the others were all retrospectively designed. Manual segmentation and multicenter studies were performed by 69% and 46% of the included studies, respectively. 3D-Slicer and MATLAB software were most commonly used for the segmentation of lesions and extraction of features. The peritumoral region was most frequently defined as dilated from the tumor boundary of 15 mm, 20 mm, or 30 mm. The median RQS of the studies was 13 (range 4–19), while all of included studies were assessed as having a high risk of bias (ROB) overall.

**Conclusions:**

Peritumoral radiomics features based on CT images showed promise in predicting the prognosis of NSCLC, although well-designed studies and further biological validation are still needed.

**Key Points:**

*• Peritumoral radiomics features based on CT images are promising and encouraging for predicting the prognosis of non-small cell lung cancer.*

*• The peritumoral region was often dilated from the tumor boundary of 15 mm or 20 mm because these were considered safe margins.*

*• The median Radiomics Quality Score of the included studies was 13 (range 4–19), and all of studies were considered to have a high risk of bias overall.*

**Supplementary Information:**

The online version contains supplementary material available at 10.1007/s00330-022-09174-8.

## Introduction

Non-small cell lung cancer (NSCLC) is the most common type of lung cancer, accounting for 85% of all cases [1, 2]. The precise survival risk stratification of patients with NSCLC is a crucial step in treatment. Although the tumor, node, and metastasis (TNM) classification for lung cancer is the most objective and authoritative indicator of the prognosis, those in identical tumor stages still have heterogeneous prognoses [3–5]. To improve the management of NSCLC and make proper treatment decisions, numerous studies have reported other independent clinical prognostic factors, including age, sex, and performance status [6–8]. In addition, medical imaging, such as CT, can also derive potential markers of prognosis, including tumor volume, pleura effusion, and radiomics [9–14].

Radiomics based on medical imaging can assess the tumor and its environment in its entirety, which can provide additional information for predicting cancer outcomes [15–17]. Several studies have successfully applied intratumor radiomics features to predict the overall survival, the prognosis of cancer recurrence, and time to progression in patients with NSCLC [17–19]. Other studies have investigated the clinical use of quantifying peritumoral regions at CT to help predict tumor invasiveness, tumor spread through air spaces, and especially prognostic outcomes [20–23]. For example, Wang et al found that the combination of radiomics features extracted from intra- and peritumoral areas could enhance the accurate prognosis prediction of pure-solid NSCLC [23]. However, the added value of extratumoral radiomics and the quality of the studies have not been systematically assessed to further explore the potential association between peritumoral radiomics features and prognosis in NSCLC.

Therefore, the aim of this study was to systematically review and appraise the results from published studies that examined the prognostic value of CT-based peritumoral radiomics features in NSCLC patients, and the potential biological underpinnings were also summarized.

## Materials and methods

This systematic review was reported in accordance with the Preferred Reporting Items for Systematic Reviews and Meta-Analysis (PRISMA) guidelines [24]. The review was registered on PROSPERO before initiation (registration no. CRD42022322916).

### Search strategy

The PubMed, Embase, Web of Science, and Cochrane Library databases were comprehensively searched up to February 21, 2022, to identify studies that used CT-based peritumoral radiomics to evaluate the prognosis in patients with NSCLC. The reference lists of the included articles and the relevant literature were also manually searched. The following basic search terms were used: NSCLC, pulmonary nodule, CT, radiomics, peritumoral, and prognosis. The detailed search criteria are described in the supplementary material. The retrieval was performed without language and date restrictions.

### Study selection

Original research articles will be included in the study. Eligibility criteria included the following: (1) patients with NSCLC; (2) evaluating the prognosis of patients by a peritumoral radiomics approach on CT. Studies were excluded if they (1) were case studies, editorials, letters, review articles and conference abstracts; (2) were not in the field of interest; or (3) were overlaps in study populations.

### Data extraction

Data to be extracted will include the following: (1) study details: first author, publication year, country, study design; (2) patient details: the source of data acquisition (single-center/multicenter), type of cohort, sample size, TNM staging, histological subtype, type of treatment, prognostic outcome; (3) imaging details: CT tube voltage, reconstruction slice thickness (mm), plain or contrast CT; (4) radiomics details: segmentation software, segmentation method, peritumoral definition, and reference, feature extraction software, type of radiomics features, number of radiomics features, radiomics feature selection methods, type of models constructed, final classifier, number of radiomics features in the final model, type of radiomics features in the final model, and performance of the models. Two independent reviewers (L.W. and C.G.) completed the initial screening and extracted data from all enrolled studies.

### Risk of bias assessment

The methodological quality of each study was evaluated by using the Radiomics Quality Score (RQS) [25] and the Prediction Model Risk of Bias Assessment Tool (PROBAST) [26]. The RQS provides a standardized and quantitative evaluation criterion for the methodology of radiomics researches. The RQS assessment contains sixteen key components from data selection, medical imaging, feature extraction, and exploratory analysis to modelling. Each item contributes to the final score and the total score ranges from -8 to 36 points [25]. Detail description of each item of RQS and the corresponding scores is provided in Table S1. PROBAST is a tool to assess the risk of bias (ROB) and the application of prediction models for diagnosis or prognosis. The risk of bias assessment of all enrolled studies was made by two reviewers (L.W. and C.G.) with a consensus agreement.

## Results

### Literature search and data extraction

**Fig. 1:**
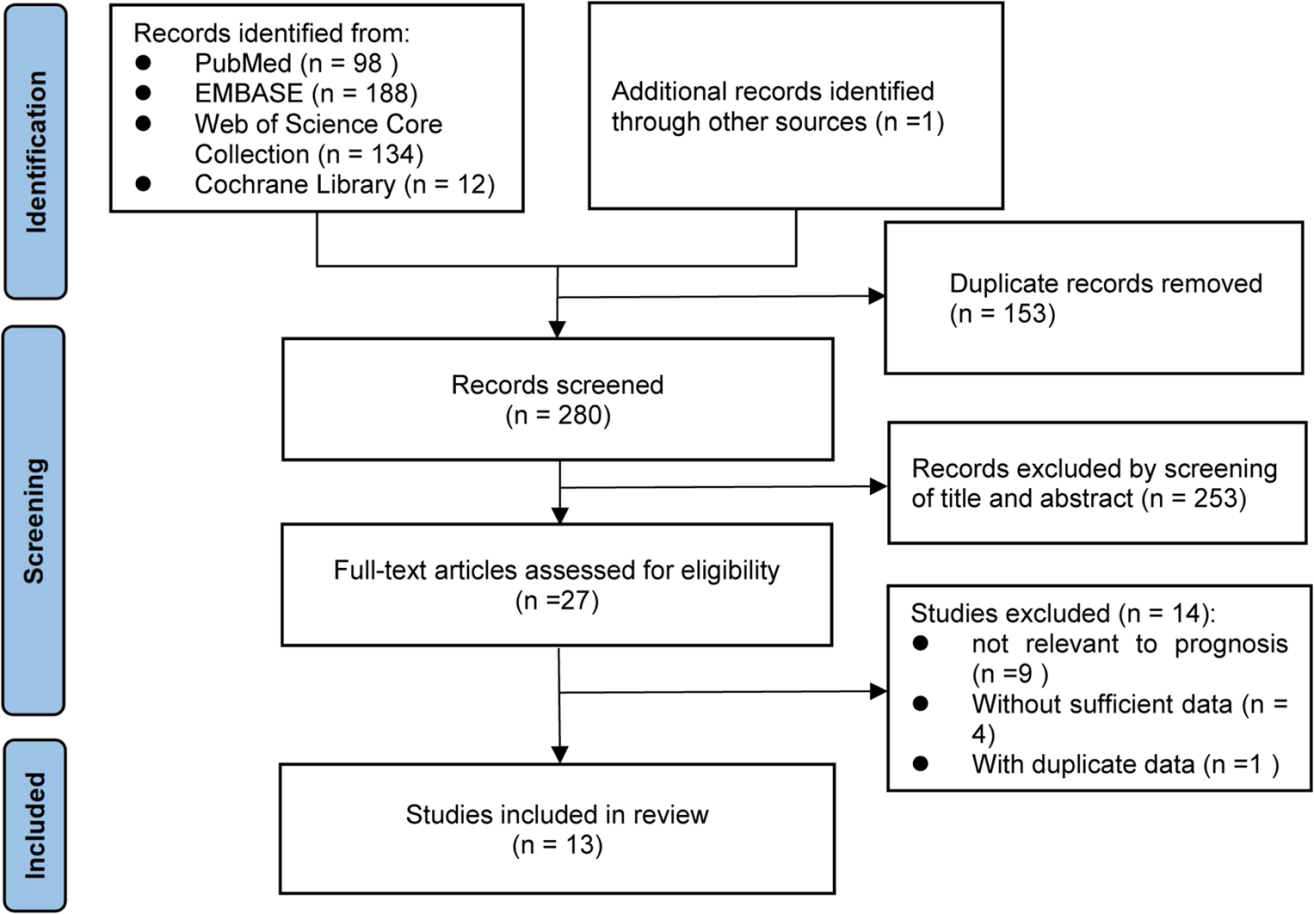
Flowchart of the study screening and selection process of this systematic review

The flow diagram of the literature search of the Preferred Reporting Items for Systematic Reviews and Meta-Analysis is shown in Fig. 1. A total of 433 studies were identified, in which 432 studies were identified by the comprehensive literature search and one study was identified by a hand search of the relevant literature. After screening and evaluating, 13 studies with 2942 patients meeting the criteria were included in this systematic review [22, 23, 27–37].

### Patient and study characteristics

The patient characteristics of 13 studies are summarized in Table 1. The included studies were published from 2017 to 2022. Almost all the studies (12/13, 92%) were retrospectively designed [22, 23, 27–36], except one of the studies, which was prospective [37]. Patients from seven studies (7/13, 54%) were from one center [22, 28–30, 34, 36, 37], and the others (6/13, 46%) were from multiple center [23, 27, 31–33, 35]. Most studies (10/13, 77%) included a training cohort and validation/test cohort, in which six studies conducted external tests from another center [23, 27, 31–33, 35]. The number of patients included in the studies ranged from 90 to 592. The type of treatment and type of prognostic outcome are summarized in Table 1. The type of treatment varied, such as surgery, adjuvant chemo-/radiotherapy, and immune checkpoint treatment. The prognostic outcome included prediction of survival [23, 27–29, 31–35, 37], distant metastasis [22, 30, 36] and response status [28, 29, 31, 34]. The most frequent study purpose was the prediction of overall survival [27, 28, 29, 31,32, 34, 37] (7/13, 54%).

**Table 1:** Basic characteristics and CT scanner information of the included studies

Study ID	Country	Study design	Institution	Training cohort	Validation/test cohort	No. patient	Type of patients	Type of Treatment	Prognostic outcome	kVp	Reconstruction slice thickness (mm)	Contrast enhanced CT
Tunali, 2017 [27]	USA	R	two	61	47 (external)	108	Stage I–IV lung adenocarcinoma	Surgical resection	OS	120 or 130 to140	1.5 or 2 or 2.5 or 3	Yes
Dou, 2018 [22]	USA	R	one	100	100 (internal)	200	Overall stage II-III lung adenocarcinoma	cCRT or trimodality (surgery + chemoradiation) or other except SBRT	Time to DM	120 or 140	2.5 or 3.75 or 5	Yes
Khorrami, 2019 [28]	USA	R	one	45	45 (internal)	90	Stage III NSCLC	Neoadjuvant chemoradiation followed by surgical resection	OS; DFS; MPR	110 to 140	0.6 to 5	--
Khorrami, 2019 [29]	USA	R	one	53	72 (internal)	125	Stage IIIb or IV NSCLC	Front-line platinum-based chemotherapy	TTP; OS; RTC	--	1 to 5	No
Antonoli, 2020 [30]	Italy	R	one	--	--	124	Stage IA to IIB NSCLC	Surgically or adjuvant chemo-/radiotherapy after surgery	TR (LR + DM); LR; DM	120	2.5	Yes
Khorrami, 2020 [31]	USA	R	two	50	62 (internal); 27 (external)	139	Advanced NSCLC	PD1/PD-L1 inhibitor	OS; Rs	100 to 120	1 to 5	Yes
Pérez-Morales, 2020 [32]	USA	R	two*	161	73 (internal); 62 (external)	296	Stage I-IV lung cancer; lung cancer by surgical resection	Surgical, Chemotherapy/Other, Radiation therapy	OS; PFS	--	--	--
Vaidya, 2020 [33]	USA	R	Three^#^	329	114 (external); 82 (external from TCIA)	525	Stage I, II resectable NSCLC	Surgery and/or adjuvant chemotherapy	DFS	--	< = 5	Yes/No^^^
Vaidya, 2020 [34]	USA	R	one	30	79 (internal)	109	Advanced NSCLC	Monotherapy with PD1/PD- L1 inhibitor	OS; HPs	--	< = 5	Yes/No^^^
Liu, 2022 [35]	China	R	two	142	62 (internal); 130 (external)	334	Clinical stage I solid lung adenocarcinoma	--	DFS	120 or 100	1 or 1.3 or 2	Yes
Davey, 2021 [36]	UK	R	one	--	--	203	Stage I and IIA NSCLC	SBRT	DF	120	3	Yes
Amico, 2020 [37]	Italy	P	one	--	--	97	Stage III NSCLC	cCRT	OS	140	3	--
Wang, 2022 [23]	China	R	Two^&^	381	163 (internal); 48 (external from sdata)	592	Clinical stage and pathologic stage IA pure-solid NSCLC	Complete surgical resection	RFS	120	1	--

*: NLST + one center; #: two centers + TCIA database; &: one center + radiogenomics data set; ^: with either contrast-enhanced CT or non-contrast CT; --: not mentioned

*cCRT*, concurrent chemoradiotherapy; *DF*, distant failure; *DFS*, disease-free survival; *DM*, distant metastasis; *HPs*, identify hyperprogressors from other response patterns; *IQR*, interquartile range; *LR*, local recurrence; *MPR*, major pathological response; *NLST*, National Lung Screening Trial; *NSCLC*, non-small cell lung cancer; *OS*, overall survival; *P*, prospective; *PFS*, progression-free survival; *R*, retrospective; *RFS*, recurrence-free survival; *Rs*, discrimination of responders from nonresponders; *RTC*, response to chemotherapy; *SBRT*, stereotactic ablative radiotherapy; *TTP*, time to progression; *TR*, total recurrence; *95% CI*, 95% confidence interval

### Radiomics workflow

The details of the acquisition parameters of the images in the radiomics studies are shown in Table 1. The slice thickness of CT ranged from 0.6 to 5 mm in most studies except for one study in which the thickness was not mentioned [32]. Some studies (6/13, 46%) conducted radiomics using contrast-enhanced CT images [22, 27, 30, 31, 35, 36] while only one study used non-contrast CT images [29]. Two studies (2/13, 15%) conducted radiomics using either contrast-enhanced or non-contrast CT images for further analysis [33, 34]. The other studies (4/13, 31%) did not mention it [23, 28, 32, 37].

The study details of the radiomics workflow, including region of interest (ROI) segmentation, feature extraction and selection, and model construction, are summarized in Table 2. The ROI segmentation was manual in most studies (9/13, 69%) [22, 23, 28, 29, 31, 33–35, 37], semi-automatic in three studies [27, 30, 32], and automatic in one study [36]. The most commonly used software for ROI segmentation was 3D-Slicer (5/13, 38%), and the most commonly used software for radiomics feature extraction was MATLAB (8/13, 62%) in the included studies (Table 2). The types of extracted radiomics features included texture features and/or first-order statistics and shape features [22, 23, 28–37]. Moreover, some novel radiomics features were introduced in the studies. For example, Tunali et al generated radial gradient and radial deviation features that represent voxel-by-voxel gradient changes [27]. And Vaidya et al analyzed radiomics features of quantitative vessel tortuosity that represent the curvedness of tumor vessels [34].

**Table 2:** Segmentation, extraction, selection methods, radiomics features, and prediction models of the included studies

Study ID	Software (segmentation; features extraction)	Segmentation method	Type of Radiomics features	No. Radiomics features extracted	Radiomics features selection methods	Final radiomics model	Type of survival models constructed	No. Radiomics features in the final survival model	Type of radiomics features in the final survival model	The models with the best performance	The corresponding performance
Tunali, 2017 [27]	Definiens Developer XD; MATLAB	Semi-automatic	Radial gradient; radial deviation	48	CCC; Pearson’s correlation analysis；Univariable analyses; Multivariable analyses	Multivariable Cox regression	2 (radiomics model; clinical + radiomics model)	2	Radial deviation outside-border separation SD; radial gradient outside-tumor separation mean	--	--
Dou, 2018 [22]	Eclipse, Varian Medical Systems and SimpleITK toolbox; PyRadiomics (Python)	Manual	First-order statistics; shape; texture	2175	ICC；mRMR；Univariable analyses; Multivariable analyses	Multivariable Cox regression	3 (clinical model; clinical + tumor radiomics model; clinical + rim radiomics model)	2	LoG 1.5mm 3D GLRLM RunEntropy; Wavelet LHL NGTDM complexity (tumor rim radiomics model)	Clinical + rim radiomics model (DM)	C-index 0.65 (test) (DM)
Khorrami, 2019 [28]	3D-Slicer; MATLAB	Manual	Texture (Haralick, LBP; HOG; CoLlAGe; Law; Gabor); shape; first-order statistics	1542	ICC; mRMR; multivariable Cox regression	Multivariable Cox regression	2 (radiomics model; clinical-pathological + radiomics model)	4 (OS); 7 (DFS)	(Peritumoral Laws, intratumoral low frequency Gabor, tumor area, and intratumoral Law_Laplacian) (OS); (peritumoral Law_Laplacian, intratumoral Laws, intratumoral Haralick inertia, intratumoral Law_Laplacian, and intratumoral median frequency Gabor) (DFS)	Clinicopathologic + radiomics model (MPR); radiomics model (OS); radiomics model (DFS)	AUC 0.90 (training), 0.86 (test) (MPR); C-index 0.84 (training) (OS); C-index 0.78 (training) (DFS)
Khorrami, 2019 [29]	--; MATLAB	Manual	Laws energy measures; Laws Laplacian; Gabor; Haralick; LBP; HOG; CoLlAGe; 3D Shape	1542	ICC；mRMR；LASSO Cox regression model	LASSO Cox regression	2 (radiomics model; clinical-pathologic + radiomics model)	6(TTP); 4(OS)	(The intratumoral Haralick correlation, Haralick diff_entropy, and intratumoral Collage diff_entropy features) (OS); the intratumoral Laws features, the peritumoral Laws features (TTP)	Radiomics model (RTC); radiomics model (TTP); clinical-pathologic + radiomics model (OS)	AUC 0.82 (training), 0.77 (test) (RTC); C-index 0.86 (training) (TTP); C-index 0.77 (training) (OS)
Antonoli, 2020 [30]	Eclipse, Varian Medical Systems; Moddicom	Semi-automatic	Morphological; first-order statistics; texture; fractal-based	94	Mann-Whitney U test；“fscaret” R package；Cox proportional-hazard model	Multivariable Cox regression	3 (radiomics model; clinicopathological model; clinicopathological + radiomics model)	4(TR);5(LR); 5(DM)	szm.glnu.norm.peritomor; stat.90thpercentile.gtv; cm.inv.var.peritumor; morph.pca.flatness.peritumor (TR)；szm.glnu.norm.peritumor；rlm.rl.entr.gtv；zsm.z.perc.gtv；morph.av.gtv；stat.var.gtv(LR)；cm.inv.var.peritumor；rlm.srhge.gtv；least.gtv；rlm.lrhge.gtv；rlm.sre.gtv(DM)	Clinicopathological + radiomics model (LR; DM; TR)	AUC 0.750 (LR); AUC 0.759 (DM); AUC 0.760 (TR)
Khorrami, 2020 [31]	3D-Slicer; MATLAB	Manual	Texture (Laws energy; Gabor; Haralick; CoLlAGe); first-order Statistics; shape	618	ICC; WLCX; LDA；unsupervised clustering analysis；univariable analysis; multivariable analysis	Multivariable Cox regression	2 (DelRADx model; Clinicopathological + DelRADx model)	8	Haralick (per texture correlation increase); Haralick (per entropy increase); Laws (per heterogeneity increase)	DelRADx model (Rs); Clinicopathological + DelRADx model (OS)	AUC 0.88 (training), 0.85 (test 1), 0.81 (test 2) (Rs); C-index 0.72 (training), 0.69 (test 1), 0.68 (test 2) (OS)
Pérez-Morales, 2020 [32]	LuTA platform; toolboxes created in MATLAB and C+ +	Semi-automatic	--	264	Univariable analysis; Pearson’s correlation analysis; multivariable analysis；CART	CART	2 (radiomics model; clinical-pathological + radiomics model)	2	NGTDM Busyness (peritumoral); Statistical Root Mean Square (intratumoral)	Clinical-pathological + radiomics model (PFS; OS)	C-index 0.81 (training), 0.80 (test) (PFS); C-index 0.83 (training), 0.81 (test) (OS)
Vaidya, 2020 [33]	3D-Slicer; MATLAB	Manual	Texture (Gabor; Haralick; Collage; Laws; Laplace); first-order statistics	4464	ICC; LASSO Cox regression model	LASSO Cox regression	2 (radiomics model; clinical-pathological + radiomics model)	13	CoLlAGe_peritumoral; Haralick_introtumoral	Clinicopathologic + radiomics model (DFS)	C-index 0.74 (training)(DFS)
Vaidya, 2020 [34]	3D-Slicer; MATLAB	Manual	Texture (Gabor; Haralick; Laws; Laplace; Collage); QVT	4538	ICC; unsupervised clustering analysis; supervised classifier (mRMR; RF/ LDA/ DLDA/ QDA/ SVM)	RF classifier^*^	5 (radiomics model)	3	Gabor feature family_5–10 mm peritumoral; QVT7_mean_peritumoral; QVT44_Entropy_Introtumoral	Radiomics model (HPs)	AUC 0.85 (training) (HPs)
Liu, 2022 [35]	ITK-SNAP; IFoundry (GE Healthcare)	Manual	first-order statistics; shape; texture (GLCM; GLRLM); deep learning (VGG-19)	5309	Univariable Cox regression, LASSO Cox regression, correlation coefficient analysis, stepwise regression, multivariable Cox regression	Multivariable Cox regression	11 (clinical model; pathological model; radiomics models; clinical-pathologic + radiomics model)	10 (PTV-3~+3)	Eight VGG19_deep learning based; first-order maximum; first-order interquartile range (PTV−3~+3)	Clinicopathologic + PTV -3~ 3 radiomics model (DFS)	AUC 0.85 (training), 0.83 (test1), 0.83 (test 2) (DFS)
Davey, 2021 [36]	In-house, validated method; Pyradiomics (Python)	Automatic	First order statistics, texture (GLCM, GLSZM, GLRLM, NGTDM, GLDM)	372	Unsupervised (Spearman rank correlation analysis; Pearson correlation analysis); Supervised (univariable Cox regression/multivariable Cox regression/ mRMR)	Multivariable Cox regression	13 (1 clinical model; 12 clinical + radiomics models)	4	GLDM dependence variance_introtumor; ‘LDLGLE’ of GLDM_peritumor; ‘Small Dependence High GrayLevel Emphasis’ of GLDM_peritumor; GLCM MCC_peritumor	Clinical + radiomics model (DF)	C-index 0.77 (DF)
Amico, 2020 [37]	--; MATLAB	Manual	First-order statistics; texture (3D GLCM; TOP-LBP)	--	RFE:(AdaBoost; CART DT; RF; XGB); the single/total ranking strategy	AdaBoost; CART DT; RF; XGB	24 (radiomics models)	9 (AdaBoost_CTV)	U 3D LBP kurtosis; 3D LBP energy; RI 3D LBP maxAss; 3D LBP energy around maxAss; RI 3D LBP energy; U 3D LBP energy around maxRel; U 3D LBP entropy; U 3D LBP skewness; inverse GLCM (*−*1,*−*1,0)	AdaBoost_CTV radiomics model (OS)	AUC 0.83 (OS)
Wang, 2022 [23]	3D-Slicer; PyRadiomics (Python)	Manual	Shape; first-order statistics; GLCM; GLSZM; GLRLM; NGTDM; GLDM; wavelet transform filter	2163	ICC; Correlation analysis; Univariable Cox regression analysis; variable clustering analysis; LASSO Cox regression	LASSO Cox regression	3 (clinical model; radiomics model; clinical + radiomics model)	18	Eight intratumoral 3D ROI features, five intratumoral 2D ROI features, five peritumoral features (Sum Entropy; Correlation; Complexity; Gray Level Variance; Gray Level Nonuniformity Normalized)	Clinical + radiomics model (RFS)	C-index 0.81 (training), 0.77 (test 1), 0.75 (test 2) (RFS)

*AUC*, the area under the receiver operating characteristic curve; *C-index*, concordance index; *CART*, Classification And Regression Tree; *CART DT*, CART Decision Tree; *CoLlAGe*, co-occurrence of local anisotropy gradient orientations; *CTV*, clinical target volume; *DelRADx*, radiomics base on the percent change of feature statistics between baseline and post- 6–8 week scans; *DFS*, disease-free survival; *DLDA*, diagonal linear discriminant analysis; *DM*, distant metastasis; *GLCM*, gray level co-occurrence matrix; *GLDM*, gray level dependence matrix; *GLRLM*, gray-level run length matrix; *GLSZM*, gray-level size zone matrix; *HOG*, histogram of oriented gradient; *HPs*, distinguishing hyperprogressors from other responsers; *ICC*, intraclass correlation coefficient; *LBP*, local binary pattern; *LDA*, linear discriminant analysis; *LR*, local recurrence; *MPR*, major pathological response; *NGTDM*, neighboring gray tone difference matrix; *OS*, overall survival; *PTV*, peritumoral volume; *QDA*, quadratic discriminant analysis; *QVT*, quantitative vessel tortuosity; *RF*, random forest; *RFE*, recursive feature elimination; *Rs*, discrimination of responders from nonresponders; *RTC,* response to chemotherapy; *SD*, standard deviation; *SVM*, support vector machine; *TR*, total recurrence; *TTP*, time to progression; *WLCX*, Wilcoxon rank sum; *XGB*, XGBoost; --: not mention; *: the prognosis outcome is to identify hyperprogressors from other responsers

The number of extracted radiomics features ranged from 48 to 5309. The radiomics feature selection methods frequently included intraclass correlation coefficients, univariable analyses, and multivariable analyses. The types of models constructed in these studies ranged from 2 to 24. The model constructed in the final radiomics model was usually a multivariable Cox model [22, 27, 28, 30, 31, 35, 36]. The number of radiomics features in the final radiomics model ranged from 2 to 18.

### The peritumoral radiomics model and possible biological underpinnings

**Fig. 2:**
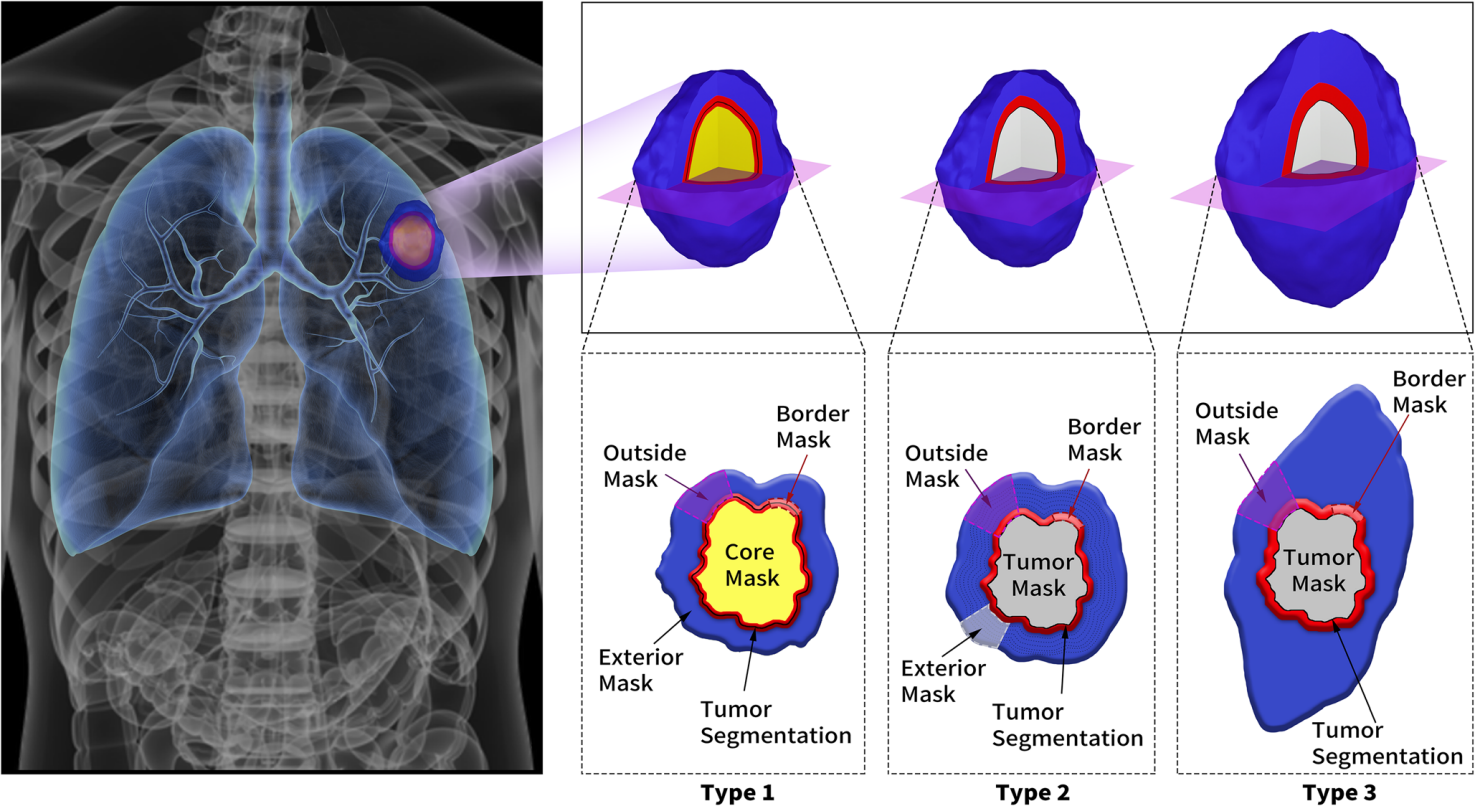
The three different types of definitions for peritumoral regions were as follows: Type 1: Border Mask: (−12.5 or −15 to + 7.5 or +10) mm [27], (−3 to + 3) mm [22, 35, 36]; Outside Mask: (0 to +17.5 or + 22.5) mm [27], (0 to + 3 or + 6) mm [35]; Exterior Mask: (+3 to +9) mm [22]. Type 2: Border Mask: (0 to +3) mm [32]; Outside Mask: (0 to +15) mm [23, 28, 29, 33, 34], (0 to +20) mm [30], (0 to +30) mm [31]. Type 3: Tumor Mask: gross tumor volume; Border Mask: clinical target volume minus Tumor Mask; Outside Mask: planning target volume minus Tumor Mask [37]. −: inward erosion; +: outward dilation; 0: tumor boundary

All the included studies segmented both the intra- and peri-tumoral regions; however, the definitions of peritumoral regions varied. Three different definitions for peritumoral regions were summarized in Fig. 2. Almost all the performances of erosion and dilation were based on the morphology of tumors and can be classified into three types. In type 1, the border mask was defined to be inward erosion 12.5/15 mm [27] or 3 mm [22, 35, 36] to outward dilation 7.5/10 mm [27] or 3 mm [22, 35, 36] along the tumor border. The outside mask was defined as an area expanding outside from the tumor to 17.5/22.5 mm [27] or 3/6 mm [35]. The exterior mask was defined as an area 3 to 9 mm away from the tumor [22]. In type 2, the border mask was defined to be the region that expands 3 mm away from the tumor boundary [32] while the criteria for the outside mask was 15 mm [23, 28, 29, 33, 34] or 20 mm [30] or 30 mm [31]. In type 3, the gross tumor volume equalled the original volume of the tumor lesion without any erosion or dilation performance. The clinical target volume contained gross tumor volume plus an area expanding outside from tumor boundary. The planning target volume was defined as the combination of tumor volume and the area dilated from the tumor border, which was necessary to manage internal motion and set-up reproducibility [37].

Moreover, the references of peritumoral region researches applied also varied in these included studies. The peritumoral regions were often dilated from the tumor boundary of 15 mm, 20 mm, or 30 mm (7/13, 54%) [23, 28–31, 33, 34]. Some of them (6/13, 46%) referred to previous findings, where a resection margin > 15 mm did not decrease the risk of recurrence or a resection margin ≥ 20 mm was the safe margin [28–31, 33, 34]. An area outside the border of the tumor was chosen as the peritumoral region in several studies (4/13, 31%), where microscopic extension of cancerous islets or “real invasive front” can still be found [22, 35–37].

Several researchers have explored the biological underpinnings of peritumoral radiomics features in the prediction of the prognostic outcome of patients with NSCLC [27, 31–33]. Khorrami et al investigated associations between changes in radiomics features and the density of tumor-infiltrating lymphocytes on digitized hematoxylin-eosin images [31]. Pérez-Morales et al analyzed the associations between the final two radiomics features with gene probesets [32]. Vaidya et al investigated associations between prognostic radiomics features and tumor-infiltrating lymphocytes (radiopathomic analysis), as well as the radiomics features and mRNA sequencing data (radiogenomic analysis) [33]. Tunali et al explored potential biological underpinnings by analyzing the correlations of radiomics features with semantic radiological features [27]. Others also discussed the possible pathological basis of prognostic radiomics features from the peritumoral region, such as “real invasive front,” hypoxic tumor environment, neovascularization and angiogenesis in the tumor microenvironment, lymphovascular tumor invasion and micrometastasis [22, 28–30, 34, 35].

### The performance of the models

The models with the best performance and the corresponding performance metrics in the included studies were summarized in Table 2. The concordance index (C-index) and the area under the receiver operating characteristic curve (AUC) were used to evaluate the performance of these models in twelve of thirteen of included studies [22, 23, 28–37]. The peritumoral radiomics features played an important role in the survival models [22, 23, 27–37]. The values of C-index or AUC of these best-performance models ranged from 0.65 to 0.90 [22, 23, 28–37].

### Quality assessment

**Fig. 3:**
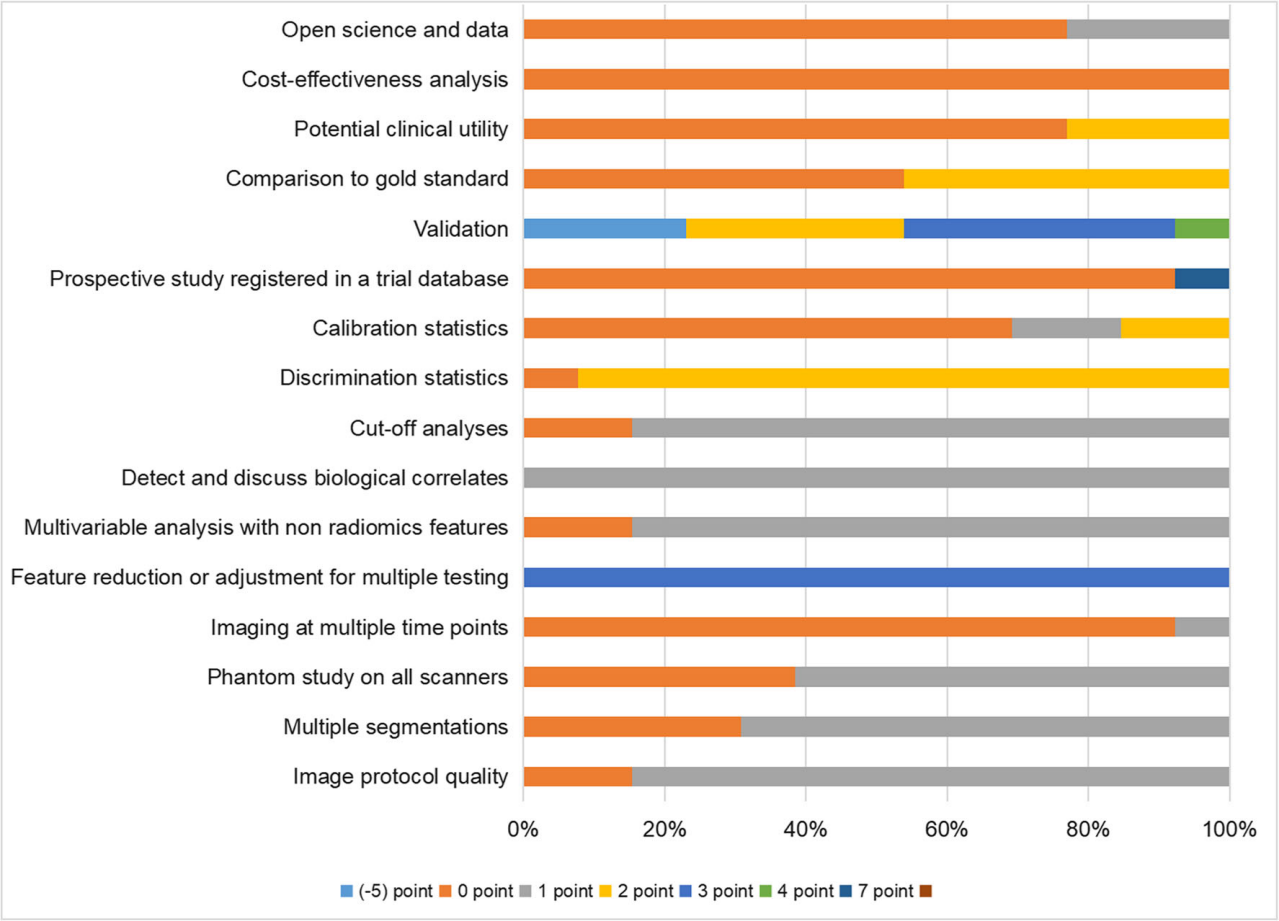
Quality assessment of included studies by the Radiomics Quality Score (RQS) and presenting the percentages of scores of the included studies

**Fig. 4:**
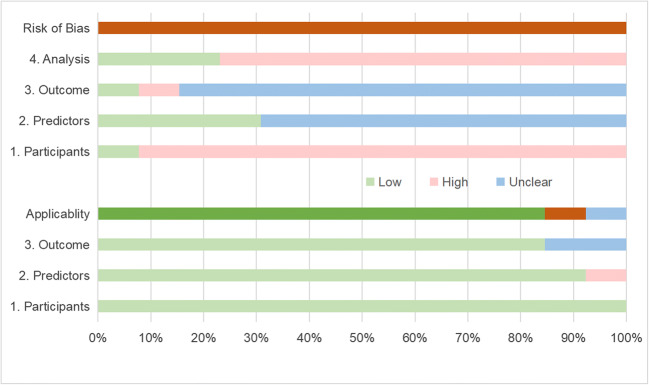
The percentage of the included studies rated by the risk of bias and applicability using the Prediction Model Risk of Bias Assessment Tool (PROBAST)

The total RQS and the percentages of the maximum score are summarized in Table 3. The median RQS of the studies was 13 (range 4–19), and the corresponding percentage of the score was 36.11% (range 11.11–52.78%). Figure 3 shows the percentages of scores in the studies for the sixteen components of RQS. The results of the ROB and the applicability assessments of these studies were presented in Table 4. Figure 4 presents the percentage of the studies rated by level of concern, ROB, and applicability for each domain. All of studies were assessed as high ROB overall [22, 23,27–32, 33–37]. Most studies (12/13, 92%) were considered low concern regarding the applicability [22, 23, 28–33, 35–37].

**Table 3:** Radiomics quality scores for the included studies

Study ID	Image protocol quality (0–2)	Multiple segmentations (0 or 1)	Phantom study on all scanners (0 or 1)	Imaging at multiple time points (0 or 1)	Feature reduction or adjustment (−3 or 3)	Non radiomics features (0 or 1)	Biological correlates (0 or 1)	Cut-off analyses (0 or 1)	Discrimination statistics (0–2)	Calibration statistics (0–2)	Prospective study (0 or 7)	Validation (−5 to 5)	Comparison to gold standard (0 or 2)	Pontential clinical utility (0 or 2)	Cost-effectiveness analysis (0 or 1)	Open science and data (0–4)	Total points (−12 to 36) (%)
Tunali, 2017 [27]	1	0	1	0	3	1	1	1	0	0	0	3	2	0	0	0	13 (36.11)
Dou, 2018 [22]	1	1	1	0	3	1	1	1	2	0	0	2	2	0	0	2	17 (47.22)
Khorrami, 2019 [28]	1	1	1	0	3	1	1	1	2	0	0	2	0	2	0	0	16 (44.44)
Khorrami, 2019 [29]	0	1	1	0	3	1	1	1	2	1	0	2	0	2	0	0	15 (41.67)
Antonoli, 2020 [30]	1	1	0	0	3	1	1	1	2	2	0	-5	2	0	0	1	17 (47.22)
Khorrami, 2020 [31]	1	0	1	0	3	1	1	1	2	0	0	3	0	0	0	1	16 (44.44)
Pérez-Morales, 2020 [32]	0	1	1	0	3	1	1	1	2	0	0	3	0	0	0	0	14 (38.89)
Vaidya, 2020 [33]	1	0	1	0	3	1	1	1	2	2	0	4	2	0	0	1	19 (52.78)
Vaidya, 2020 [34]	1	1	1	0	3	0	1	1	2	0	0	2	0	0	0	0	12 (33.33)
Liu, 2022 [35]	1	1	0	0	3	1	1	1	2	0	0	3	2	0	0	1	19 (52.78)
Davey, 2021 [36]	1	0	0	1	3	1	1	0	2	0	0	-5	0	0	0	1	7 (19.44)
Amico, 2020 [37]	1	1	0	0	3	0	1	0	2	0	7	-5	0	0	0	0	11 (30.56)
Wang, 2022 [23]	1	1	0	0	3	1	1	1	2	1	0	3	2	2	0	1	19 (52.78)

**Table 4:** Prediction model risk of bias assessment of included studies (PROBAST)

Study ID	Risk				Applicability			Overall	
	Participants	Predictors	Outcome	Analysis	Participants	Predictors	Outcome	ROB	Applicability
Tunali, 2017 [27]	-	+	-	-	+	-	?	-	-
Dou, 2018 [22]	-	?	?	-	+	+	+	-	+
Khorrami, 2019 [28]	-	+	?	-	+	+	+	-	+
Khorrami, 2019 [29]	-	+	+	-	+	+	+	-	+
Antonoli, 2020 [30]	-	?	?	+	+	+	+	-	+
Khorrami, 2020 [31]	-	?	?	-	+	+	+	-	+
Pérez-Morales, 2020 [32]	-	?	?	-	+	+	+	-	+
Vaidya, 2020 [33]	-	?	?	+	+	+	+	-	+
Vaidya, 2020 [34]	-	?	?	-	+	+	?	-	?
Liu, 2022 [35]	-	?	?	-	+	+	+	-	+
Davey, 2021 [36]	-	?	?	-	+	+	+	-	+
Amico, 2020 [37]	+	+	?	-	+	+	+	-	+
Wang, 2022 [23]	-	?	?	+	+	+	+	-	+

*ROB*, risk of bias; *PROBAST*, prediction model risk of bias assessment tool; *+* indicates low ROB/low concern regarding applicability; − indicates high ROB; ? indicates unclear ROB/unclear concern regarding applicability

## Discussion

In this systematic review, we found that the radiomics features extracted from the peritumoral lung parenchyma on CT images can be considered a potential prognostic factor for patients with NSCLC. However, the included studies showed considerable variability and heterogeneity (including CT acquisition parameters and radiomics methodology) in each step of radiomics analysis.

Using standardized radiomics analysis was advocated to eliminate unnecessary confounding variability [25, 38]. With included studies having a wide range of section thicknesses (0.6–5 mm), the impact of section thickness on the performance of the model should be evaluated. Khorrami et al evaluated the impact of section thickness on the performance of the classifier and found that the areas under the receiver operating characteristic curves for the radiomics model decreased slightly when the section thickness increased [28, 29, 31]. Bettinelli et al found that the agreement of seven radiomics software programs varied [39]. The test-retest and differences in the inter-CT and intra-CT protocols can affect the stability of radiomics features to different degrees [40]. Therefore, several studies selected stable and reproducible features on the test-retest RIDER lung CT dataset and retained features with an intraclass correlation coefficient of 0.75, 0.8, 0.85 or greater [22, 27–29, 31–34].

ROIs can be segmented manually or (semi)automatically. However, manual segmentation remained the main method in the radiomics studies, and 69% of included studies segmented the ROI manually [22, 23, 28, 29, 31, 33–35, 37]. The variability in manual delineations can be reduced by multiple segmentation, but it is time-consuming [25]. Hence, rapid and reliable automatic ROI segmentation is highly desired and is still challenging. Some efforts to automatically segment the lung nodules have been made, which is promising in the future [41–43]. Feature selection, modeling methodology, and validation were three major aspects of the radiomics model. Feature reduction for high-throughput radiomics features was performed to decrease the risk of overfitting by multiple methodologies, such as max-relevance and min-redundant, the least absolute shrinkage and selection operator method [22, 28, 29, 33, 35]. Validation is an indispensable component of radiomics analysis [25]. Most of the included studies conducted internal validation or even external validation from another center [22, 23, 27–29, 31–35].

CT images may contain information that reflects the underlying pathophysiology of the tumor and that results in the conversion of images into structured data to assist in clinical decision support [38]. Peritumoral mask segmentation is usually based on morphologic operations (dilation) from the lesion boundary. Features are often extracted from three-dimension volume of interest and/or a section-by-section basis [22, 23, 27–31], while a few studies extracted from the three slices have the maximum area of the tumor [33, 34]. With an underlying biological rationale, such as “real invasive front” and micrometastasis around the tumor, the peritumoral regions of the included studies were dilated from the tumor boundary between 3 and 30 mm [22, 23, 27–36]. The biological underpinning of radiomics is significantly important to its wider use and further validation. Efforts to explain the biological meaning of radiomics are emerging, including relationships with semantic features, gene expression, microscopic histopathologic findings, and macroscopic histopathologic marker expression [44]. Encouragingly, several researchers have investigated the correlation between prognostic radiomics features and the density of tumor-infiltrating lymphocytes and gene and mRNA sequencing data [31–33]. This exploration will reinforce our understanding of the biological meaning of peri-tumoral radiomics in the predicting prognosis of NSCLC patients.

The RQS was used to assess the methodology, analysis, and reporting of a radiomics study. The median RQS of the studies was 13 (range 4–19), which indicates that most of the included studies did not reach a median level of radiomics quality. All the included studies conducted feature reduction, and biological correlates discussions. None of the included studies conducted a cost-effectiveness analysis, and most of the studies lacked open science. According to the PROBAST, all of the studies were considered to have a high ROB overall. The reasons for model development and validation studies with high ROB may be as follows: (1) Most of the included studies (12/13, 92%) were retrospective studies. (2) The calibration was not evaluated in most studies. (3) Whether predictors were assessed without knowledge of outcome information was also not mentioned.

This systematic review has several limitations that should be noted. First, the number of eligible studies was relatively small. Second, because high heterogeneity was found in radiomics analysis, such as the type of treatment, outcome of prognosis, and radiomics modeling, a meta-analysis of pooled outcomes was not conducted. Third, most of the studies were evaluated as having low RQS and high ROB, so the results should be interpreted with caution.

In conclusion, growing evidence has shown that peritumoral CT-based radiomics features in predicting the prognosis of NSCLC are promising, although they need standardization in radiomics analysis. Because most of the studies were performed retrospectively, studies based on prospective, multiple centers as well as biological correlations should be further conducted to promote their clinical use.

## Supplementary Information

ESM 1 (DOCX 32 kb)

## Abbreviations

AUC: Area under the receiver operating characteristic curve
C-index: Concordance index
NSCLC: Non-small cell lung cancer
PROBAST: Prediction model risk of bias assessment tool
ROB: Risk of bias
ROI: Region of interest
RQS: Radiomics quality score
TNM: Tumor, node, and metastasis

## Declarations

### Guarantor

The scientific guarantor of this publication is Chen Gao.

### Statistics and biometry

No complex statistical methods were necessary for this paper.

### Methodology

• retrospective

• Systematic review

## References

[1] Sung H, Ferlay J, Siegel RL (2021). Global Cancer Statistics 2020: GLOBOCAN estimates of incidence and mortality worldwide for 36 cancers in 185 countries. CA Cancer J Clin.

[2] She Y, Jin Z, Wu J (2020). Development and validation of a deep learning model for non-small cell lung cancer survival. JAMA Netw Open.

[3] Rami-Porta R, Asamura H, Travis WD, Rusch VW (2017). Lung cancer - major changes in the American Joint Committee on Cancer eighth edition cancer staging manual. CA Cancer J Clin.

[4] Zhai W, Duan F, Li D (2022). Risk stratification and adjuvant chemotherapy after radical resection based on the clinical risk scores of patients with stage IB-IIA non-small cell lung cancer. Eur J Surg Oncol.

[5] Schegoleva AA, Khozyainova AA, Fedorov AA (2021). Prognosis of different types of non-small cell lung cancer progression: current state and perspectives. Cell Physiol Biochem.

[6] Ahmed T, Lycan T, Dothard A (2020). Performance status and age as predictors of immunotherapy outcomes in advanced non-small-cell lung cancer. Clin Lung Cancer.

[7] Sachs E, Sartipy U, Jackson V (2021). Sex and survival after surgery for lung cancer: a Swedish Nationwide Cohort. Chest.

[8] Sehgal K, Gill RR, Widick P (2021). Association of performance status with survival in patients with advanced non-small cell lung cancer treated with pembrolizumab monotherapy. JAMA Netw Open.

[9] Xie HJ, Zhang X, Mo YX, Long H, Rong TH, Su XD (2019). Tumor volume is better than diameter for predicting the prognosis of patients with early-stage non-small cell lung cancer. Ann Surg Oncol.

[10] Su XD, Xie HJ, Liu QW, Mo YX, Long H, Rong TH (2017). The prognostic impact of tumor volume on stage I non-small cell lung cancer. Lung Cancer.

[11] Yoshimura A, Yamada T, Tsuji T (2019). Prognostic impact of pleural effusion in EGFR-mutant non-small cell lung cancer patients without brain metastasis. Thorac Cancer.

[12] Yang F, Zhang J, Zhou L (2022). CT-based radiomics signatures can predict the tumor response of non-small cell lung cancer patients treated with first-line chemotherapy and targeted therapy. Eur Radiol.

[13] Huang L, Chen J, Hu W (2019). Assessment of a radiomic signature developed in a general nsclc cohort for predicting overall survival of ALK-positive patients with different treatment types. Clin Lung Cancer.

[14] de Jong EEC, van Elmpt W, Rizzo S (2018). Applicability of a prognostic CT-based radiomic signature model trained on stage I-III non-small cell lung cancer in stage IV non-small cell lung cancer. Lung Cancer.

[15] Aerts HJ (2016). The potential of radiomic-based phenotyping in precision medicine: a review. JAMA Oncol.

[16] Liu Z, Wang S, Dong D (2019). The applications of radiomics in precision diagnosis and treatment of oncology: opportunities and challenges. Theranostics.

[17] Khorrami M, Bera K, Leo P (2020). Stable and discriminating radiomic predictor of recurrence in early-stage non-small cell lung cancer: multi-site study. Lung Cancer.

[18] Wang L, Dong T, Xin B (2019). Integrative nomogram of CT imaging, clinical, and hematological features for survival prediction of patients with locally advanced non-small cell lung cancer. Eur Radiol.

[19] van Laar M, van Amsterdam WAC, van Lindert ASR, de Jong PA, Verhoeff JJC (2020). Prognostic factors for overall survival of stage III non-small cell lung cancer patients on computed tomography: a systematic review and meta-analysis. Radiother Oncol.

[20] Wu L, Gao C, Ye J (2021). The value of various peritumoral radiomic features in differentiating the invasiveness of adenocarcinoma manifesting as ground-glass nodules. Eur Radiol.

[21] Liao G, Huang L, Wu S (2022). Preoperative CT-based peritumoral and tumoral radiomic features prediction for tumor spread through air spaces in clinical stage I lung adenocarcinoma. Lung Cancer.

[22] Dou TH, Coroller TP, van Griethuysen JJM, Mak RH, Aerts HJWL (2018). Peritumoral radiomics features predict distant metastasis in locally advanced NSCLC. PLoS One.

[23] Wang T, She Y, Yang Y (2022). Radiomics for survival risk stratification of clinical and pathologic stage IA pure-solid non-small cell lung cancer. Radiology.

[24] Page MJ, McKenzie JE, Bossuyt PM (2021). The PRISMA 2020 statement: an updated guideline for reporting systematic reviews. BMJ.

[25] Lambin P, Leijenaar RTH, Deist TM (2017). Radiomics: the bridge between medical imaging and personalized medicine. Nat Rev Clin Oncol.

[26] Moons KGM, Wolff RF, Riley RD (2019). PROBAST: a tool to assess risk of bias and applicability of prediction model studies: explanation and Elaboration. Ann Intern Med.

[27] Tunali I, Stringfield O, Guvenis A (2017). Radial gradient and radial deviation radiomic features from pre-surgical CT scans are associated with survival among lung adenocarcinoma patients. Oncotarget.

[28] Khorrami M, Jain P, Bera K (2019). Predicting pathologic response to neoadjuvant chemoradiation in resectable stage III non-small cell lung cancer patients using computed tomography radiomic features. Lung Cancer.

[29] Khorrami M, Khunger M, Zagouras A (2019). Combination of peri- and intratumoral radiomic features on baseline CT scans predicts response to chemotherapy in lung adenocarcinoma. Radiol Artif Intell.

[30] Akinci D'Antonoli T, Farchione A, Lenkowicz J (2020). CT radiomics signature of tumor and peritumoral lung parenchyma to predict nonsmall cell lung cancer postsurgical recurrence risk. Acad Radiol.

[31] Khorrami M, Prasanna P, Gupta A (2020). Changes in CT radiomic features associated with lymphocyte distribution predict overall survival and response to immunotherapy in non-small cell lung cancer. Cancer Immunol Res.

[32] Pérez-Morales J, Tunali I, Stringfield O (2020). Peritumoral and intratumoral radiomic features predict survival outcomes among patients diagnosed in lung cancer screening. Sci Rep.

[33] Vaidya P, Bera K, Gupta A (2020). CT derived radiomic score for predicting the added benefit of adjuvant chemotherapy following surgery in stage I, II resectable non-small cell lung cancer: a retrospective multicohort study for outcome prediction. Lancet Digit Health.

[34] Vaidya P, Bera K, Patil PD (2020). Novel, non-invasive imaging approach to identify patients with advanced non-small cell lung cancer at risk of hyperprogressive disease with immune checkpoint blockade. J Immunother Cancer.

[35] Liu K, Li K, Wu T (2022). Improving the accuracy of prognosis for clinical stage I solid lung adenocarcinoma by radiomics models covering tumor per se and peritumoral changes on CT. Eur Radiol.

[36] Davey A, van Herk M, Faivre-Finn C, Brown S, McWilliam A (2021). Optimising use of 4D-CT phase information for radiomics analysis in lung cancer patients treated with stereotactic body radiotherapy. Phys Med Biol.

[37] D'Amico NC, Sicilia R, Cordelli E (2020). Radiomics-based prediction of overall survival in lung cancer using different volumes-of-interest. Applied Sciences.

[38] Gillies RJ, Kinahan PE, Hricak H (2016). Radiomics: images are more than pictures, they are data. Radiology.

[39] Bettinelli A, Marturano F, Avanzo M (2022). A novel benchmarking approach to assess the agreement among radiomic tools. Radiology.

[40] Peng X, Yang S, Zhou L (2022). Repeatability and reproducibility of computed tomography radiomics for pulmonary nodules: a multicenter phantom study. Invest Radiol.

[41] Halder A, Chatterjee S, Dey D, Kole S, Munshi S (2020). An adaptive morphology-based segmentation technique for lung nodule detection in thoracic CT image. Comput Methods Programs Biomed.

[42] Shaukat F, Raja G, Gooya A, Frangi AF (2017). Fully automatic detection of lung nodules in CT images using a hybrid feature set. Med Phys.

[43] Wu W, Gao L, Duan H, Huang G, Ye X, Nie S (2020). Segmentation of pulmonary nodules in CT images based on 3D-UNET combined with three-dimensional conditional random field optimization. Med Phys.

[44] Tomaszewski MR, Gillies RJ (2021). The biological meaning of radiomic features. Radiology.

