# Supplementary material for: Can quantitative peritumoral CT radiomics features predict the prognosis of patients with non-small cell lung cancer? A systematic review
Source: Eur Radiol. 2022 Oct 29;33(3):2105–17. doi: 10.1007/s00330-022-09174-8 (PMC9935659; doi:10.1007/s00330-022-09174-8)
Supplement: Supplementary file 1 — (DOCX 32 kb) [file 330_2022_9174_MOESM1_ESM.docx]

**Supplementary Material**

1. **Search Strategy**

**1. PubMed search strategy (n = 98)**

#1 "Lung Neoplasms"[MeSH Terms] OR "Carcinoma, Non-Small-Cell Lung"[MeSH Terms] OR "lung neoplasm*"[Title/Abstract] OR "pulmonary neoplasm*"[Title/Abstract] OR "lung cancer*"[Title/Abstract] OR "lung tumo*"[Title/Abstract] OR "lung carcinoma*"[Title/Abstract] OR "pulmonary cancer*"[Title/Abstract] OR "pulmonary tumo*"[Title/Abstract] OR "pulmonary carcinoma*"[Title/Abstract] OR "Non-Small Cell Lung Cancer"[Title/Abstract] OR "lung adenocarcinoma*"[Title/Abstract]

#2 "Solitary Pulmonary Nodule"[MeSH Terms] OR "pulmonary nodule*"[Title/Abstract] OR "lung nodule*"[Title/Abstract] OR "ground-glass nodule*"[Title/Abstract] OR "solid nodule*"[Title/Abstract] OR "subsolid nodule*"[Title/Abstract] OR "part-solid nodule*"[Title/Abstract] OR "non-solid nodule*"[Title/Abstract]

#3 "Tomography, X-Ray Computed"[MeSH Terms] OR "tomography scanners, x-ray computed"[Title/Abstract] OR "computed tomography"[Title/Abstract] OR "computer assisted tomography"[Title/Abstract] OR "CT"[Title/Abstract]

#4 "Machine Learning"[MeSH Terms] OR "machine learning"[Title/Abstract] OR "radiomic"[Title/Abstract] OR "radiomics"[Title/Abstract] OR "texture"[Title/Abstract] OR "textures"[Title/Abstract] OR "quantitative"[Title/Abstract] OR "computer-aided"[Title/Abstract] OR "deep learning"[Title/Abstract]

#5 "peritumoral"[Title/Abstract] OR "peritumoural"[Title/Abstract] OR "peritumor"[Title/Abstract] OR "peritumour"[Title/Abstract] OR "perinodular"[Title/Abstract] OR "surrounding"[Title/Abstract] OR "peripheral"[Title/Abstract] OR "border"[Title/Abstract] OR "marginal"[Title/Abstract] OR "margin"[Title/Abstract] OR "edge"[Title/Abstract] OR "adjacent"[Title/Abstract] OR "radial"[Title/Abstract] OR "penumbra"[Title/Abstract]

#6 "prognosis"[MeSH Terms] OR "survival"[Title/Abstract] OR "recurrence"[Title/Abstract] OR "metastasis"[Title/Abstract] OR "prognostic"[Title/Abstract] OR "prognosis"[Title/Abstract]

#7 (#1 OR #2) AND #3 AND #4 AND #5 AND #6

**2.** **Embase (n = 188)**

No. Query Results Results

#17. #5 AND #9 AND #12 AND #13 AND #16 188

#16. #14 OR #15 2,974,197

#15. 'survival':ti,ab,kw OR 'prognosis':ti,ab,kw OR 2,774,566

'recurrence':ti,ab,kw OR 'prognostic':ti,ab,kw OR

'metastasis':ti,ab,kw

#14. 'prognosis'/exp 840,875

#13. 'peritumoral':ti,ab,kw OR 'peritumoural':ti,ab,kw 1,707,629

OR 'peritumor':ti,ab,kw OR 'peritumour':ti,ab,kw

OR 'perinodular':ti,ab,kw OR

'surrounding':ti,ab,kw OR 'peripheral':ti,ab,kw

OR 'border':ti,ab,kw OR 'marginal':ti,ab,kw OR

'margin':ti,ab,kw OR 'edge':ti,ab,kw OR

'adjacent':ti,ab,kw OR 'radial':ti,ab,kw OR

'penumbra':ti,ab,kw

#12. #10 OR #11 1,228,662

#11. 'machine learning':ti,ab,kw OR 1,029,441

'radiomic':ti,ab,kw OR 'radiomics':ti,ab,kw OR

'texture':ti,ab,kw OR 'textures':ti,ab,kw OR

'quantitative':ti,ab,kw OR

'computer-aided':ti,ab,kw OR 'deep

learning':ti,ab,kw

#10. 'machine learning'/exp 294,985

#9. #6 OR #7 OR #8 904,525

#8. 'computed tomography':ti,ab,kw OR 'computer 865,705

assisted tomography':ti,ab,kw OR 'ct':ti,ab,kw

#7. 'computed tomography scanner'/exp 54,930

#6. 'x-ray computed tomography'/exp 81,650

#5. #1 OR #2 OR #3 OR #4 550,254

#4. 'lung neoplasm*':ti,ab,kw OR 'pulmonary 349,122

neoplasm*':ti,ab,kw OR 'lung cancer*':ti,ab,kw OR

'lung tumo*':ti,ab,kw OR 'lung

carcinoma*':ti,ab,kw OR 'pulmonary

cancer*':ti,ab,kw OR 'pulmonary tumo*':ti,ab,kw

OR 'pulmonary carcinoma*':ti,ab,kw OR 'non-small

cell lung cancer':ti,ab,kw OR 'lung

adenocarcinoma*':ti,ab,kw OR 'pulmonary

nodule*':ti,ab,kw OR 'lung nodule*':ti,ab,kw OR

'ground-glass nodule*':ti,ab,kw OR 'solid

nodule*':ti,ab,kw OR 'subsolid nodule*':ti,ab,kw

OR 'part-solid nodule*':ti,ab,kw OR 'non-solid

nodule*':ti,ab,kw

#3. 'lung cancer'/exp 431,444

#2. 'lung nodule'/exp 24,556

#1. 'lung tumor'/exp 497,944

**3. Web of Science Core Collection search strategy (n = 134)**

#1 TS=("lung neoplasm*") OR TS=("pulmonary neoplasm*") OR TS=("lung cancer*") OR TS=("lung tumo*") OR TS=("lung carcinoma*") OR TS=("pulmonary cancer*") OR TS=("pulmonary tumo*") OR TS=("pulmonary carcinoma*") OR TS=("non-small-cell lung cancer*") OR TS=("lung adenocarcinoma*") OR TS=("pulmonary nodule*") OR TS=("lung nodule*") OR TS=("ground-glass nodule*") OR TS=("solid nodule*") OR TS=("subsolid nodule*") OR TS=("part-solid nodule*") OR TS=("non-solid nodule*")

#2 TS= ("Tomography, X-Ray Computed") OR TS= ("computed tomography") OR TS= ("computer assisted tomography") OR TS=("CT") OR TS=("CAT")

#3 TS= ("machine learning") OR TS=("radiomic*") OR TS=("textur*") OR TS=("quantitative") OR TS=("computer-aided") OR TS= ("deep learning")

#4 TS=("peritumoral") OR TS=("peritumoural") OR TS=("peritumor") OR TS=("peritumour") OR TS=("perinodular") OR TS=("surrounding") OR TS=("peripheral") OR TS=("border") OR TS=("marginal") OR TS=("margin") OR TS=("edge") OR TS=("adjacent") OR TS=("radial") OR TS=("penumbra")

#5 TS=("survival") OR TS=("recurrence") OR TS=("metastasis") OR TS=("prognostic") OR TS=("prognosis")

1. **Cochrane search strategy (n = 12)**

#1 MeSH descriptor: [Lung Neoplasms] explode all trees 8353

#2 MeSH descriptor: [Solitary Pulmonary Nodule] explode all trees 86

#3 MeSH descriptor: [Carcinoma, Non-Small-Cell Lung] explode all trees 4670

#4 ("lung neoplasm*"): ti,ab,kw OR ("pulmonary neoplasm*"):ti,ab,kw OR ("lung cancer*"):ti,ab,kw OR ("lung tumo*"):ti,ab,kw OR ("lung carcinoma*"):ti,ab,kw (Word variations have been searched) 23364

#5 ("pulmonary cancer*"): ti,ab,kw OR ("pulmonary tumo*"):ti,ab,kw OR ("pulmonary carcinoma*"):ti,ab,kw OR ("Non-Small-Cell Lung Cancer*"):ti,ab,kw OR ("lung adenocarcinoma*"):ti,ab,kw (Word variations have been searched) 13700

#6 ("pulmonary nodule*"): ti,ab,kw OR ("lung nodule*"):ti,ab,kw OR ("ground-glass nodule*"):ti,ab,kw OR ("solid nodule*"):ti,ab,kw OR ("subsolid nodule*"):ti,ab,kw (Word variations have been searched) 572

#7 ("part-solid nodule*"): ti,ab,kw OR ("non-solid nodule*"):ti,ab,kw (Word variations have been searched) 19

#8 #1 OR #2 OR #3 OR #4 OR #5 OR #6 OR #7 24054

#9 MeSH descriptor: [Tomography, X-Ray Computed] explode all trees 5401

#10 ("tomography scanners, x-ray computed"): ti,ab,kw OR ("computed tomography"):ti,ab,kw OR ("computer assisted tomography"):ti,ab,kw OR ("CT"):ti,ab,kw OR ("CAT"):ti,ab,kw 92239

#11 #9 OR #10 93222

#12 MeSH descriptor: [Machine Learning] explode all trees 182

#13 ("machine learning"): ti,ab,kw OR ("radiomic*"):ti,ab,kw OR ("textur*"):ti,ab,kw OR ("quantitative"):ti,ab,kw OR ("computer-aided"):ti,ab,kw 29479

#14 ("deep learning"): ti,ab,kw 675

#15 #12 OR #13 OR #14 29942

#16 ("peritumo*"): ti,ab,kw OR ("perinodular"):ti,ab,kw OR ("surrounding"):ti,ab,kw OR ("peripheral"):ti,ab,kw 54343

#17 ("border"): ti,ab,kw OR ("marginal"):ti,ab,kw OR ("margin"):ti,ab,kw OR ("edge"):ti,ab,kw OR ("adjacent"):ti,ab,kw 23457

#18 ("radial"): ti,ab,kw OR ("penumbra"):ti,ab,kw 5849

#19 #16 OR #17 OR #18 81617

#20 MeSH descriptor: [Prognosis] explode all trees 165236

#21 ("survival"): ti,ab,kw OR ("recurrence"):ti,ab,kw OR ("metastasis"):ti,ab,kw OR ("prognostic"):ti,ab,kw OR ("prognosis"):ti,ab,kw 182068

#22 #20 OR #21 305909

#23 #8 AND #11 AND #15 AND #19 AND #22 12

1. Supplementary Table:

Table S1. The detail description and the corresponding scores of Radiomics Quality Score (RQS) items[1].

| RQS items and points range | Description and corresponding points |
| --- | --- |
| 1. Image protocol quality (0 - 2) | Protocols are well-documented (+1) and/or public protocol is used (+1) |
| 1. Multiple segmentations (0 or 1) | Analyse feature robustness to segmentation variabilities for different physicians/algorithms/software (+1) |
| 1. Phantom study on all scanners (0 or 1) | Analyse feature robustness to different scanner /vendor (+1) |
| 1. Imaging at multiple time points (0 or 1) | Analyse feature robustness to temporal variabilities, e.g. organ movement, organ expansion/shrinkage (+1) |
| 1. Feature reduction or adjustment (-3 or 3) | Neither feature reduction or adjustment for multiple testing is implemented (-3); otherwise (+ 3) |
| 1. Non radiomics features (0 or 1) | Non radiomics features e.g. EGFR mutation is included in multivariable analysis (+1) |
| 1. Biological correlates (0 or 1) | Detect and discuss correlation between biology and radiomic features (+1) |
| 1. Cut-off analyses (0 or 1) | Determine risk groups by either the median, a previously published cut-off or report a continuous risk variable (+1) |
| 1. Discrimination statistics (0 - 2) | A discrimination statistic and its statistical significance are reported (+1) and a resampling method technique is also applied (+1) |
| 1. Calibration statistics (0 - 2) | A calibration statistic and its statistical significance are reported (+ 1) and a resampling method technique is also applied (+ 1) |
| 1. Prospective study (0 or 7) | Prospective study registered in a trial database (+ 7) |
| 1. Validation (-5 - 5) | Validation is missing (- 5); validation is based on a dataset from the same institute (+ 2); validation is based on a dataset from another institute (+ 3); validation is based on two datasets from two distinct institutes (+ 4); the study validates a previously published signature (+ 4); validation is based on three or more datasets from distinct institutes (+ 5) |
| 1. Comparison to gold standard (0 or 2) | Evaluate the model agrees with/is superior to the current “gold standard” method e.g. TNM-staging for survival prediction (+ 2) |
| 1. Potential clinical utility (0 or 2) | Report on the application of the model in a clinical setting e.g. decision curve analysis (+ 2) |
| 1. Cost-effectiveness analysis (0 or 1) | Report on the cost-effectiveness of the clinical application e.g. QALYs (+ 1) |
| 1. Open science and data (0–4) | Open source of scans (+ 1) and/or ROI segmentations (+ 1) and/or code (+ 1) and/or the calculated features and representative ROIs (+ 1) |

*RQS*: Radiomics Quality Score; *EGFR*: epidermal growth factor receptor; *TNM*: tumor, node, and metastasis; *QALYs*: quality-adjusted life-years; *ROI*: region of interest

**Reference:**

1. Lambin P, Leijenaar RTH, Deist TM, et al (2017) Radiomics: the bridge between medical imaging and personalized medicine. Nat Rev Clin Oncol. 14:749-762.
